# Effects of pretreatment with long-acting gonadotropin-releasing hormone agonists on pregnancy outcomes in patients with minimal and mild peritoneal endometriosis: A retrospective study of 274 frozen–thawed embryo transfer cycles

**DOI:** 10.1097/MD.0000000000039553

**Published:** 2024-09-06

**Authors:** Jieyu Wang, Minling Wei, Aike Xu, Songying Zhang

**Affiliations:** a Assisted Reproduction Unit, Department of Obstetrics and Gynecology, Sir Run Run Shaw Hospital, School of Medicine, Zhejiang University, Hangzhou, China; b Key Laboratory of Reproductive Dysfunction Management of Zhejiang Province, Hangzhou, China; c Zhejiang Provincial Clinical Research Center for Obstetrics and Gynecology, Hangzhou, China.

**Keywords:** clinical pregnancy rate, endometriosis, frozen–thawed embryo transfer, live birth rate, long-acting gonadotropin-releasing hormone agonists

## Abstract

To investigate the effects of pretreatment with long-acting gonadotropin-releasing hormone agonist (GnRH-a) before frozen–thawed embryo transfer (FET) on pregnancy outcomes in patients after minimal–mild (stages I–II) peritoneal endometriosis surgery. A retrospective cohort study was performed from March 2018 to May 2019. Overall, 274 patients met inclusion criteria of undergoing FET after minimal/mild peritoneal endometriosis surgery. For the FET protocol, patients were divided into 2 groups: GnRH-a plus hormone replacement therapy (HRT) (group A, n = 154) and HRT-only (group B, n = 120), with the former divided into 2 subgroups receiving 1 (group A1, n = 80) or 2 doses (group A2, n = 74) of GnRH-a. Basic characteristics and pregnancy outcomes of groups A and B and groups A1 and A2 were compared. Clinical pregnancy rate (CPR) and live birth rate (LBR) were the primary outcomes and logistic regression was used to analyze independent correlation factors. The CPR and LBR in group A were 58.4% and 50.0%, respectively, and were not significantly higher than in group B (49.2% and 40.0%; respectively, *χ^2^* = 2.339, *P* = .126 and *χ^2^* = 2.719, *P* = .099, respectively). CPR and LBR in group A1 were not significantly lower than those in group A2 (52.5% and 45.0% vs 64.9% and 55.4%, respectively; *χ^2^* = 2.420, *P* = .120 and *χ^2^* = 1.665, *P* = .197, respectively). However, group A2’s CPR and LBR were significantly higher than group B’s (64.9% and 55.4% vs 49.2% and 40.0%, respectively; *χ^2^* = 4.560, *P* = .023 and *χ^2^* = 4.375, *P* = .026, respectively). Logistic regression analysis showed that GnRH-a pretreatment (1 or 2 doses) had no significant effect on CPR and LBR compared with the HRT-only group. Patients with minimal–mild (stages I–II) peritoneal endometriosis surgery may not require GnRH-a pretreatment before FET.

## 1 . Introduction

Endometriosis is a chronic inflammatory disease caused by the planting of active endometrial cells outside the endometrium. In population-based studies, the prevalence of endometriosis in women is about 2% to 10%, while 20% to 50% of infertile women are diagnosed with endometriosis.^[1]^ Many patients with endometriosis need assisted reproduction therapy (ART). At present, the mechanism of endometriosis is not clear, and depending on the location, endometriosis can be divided into 3 phenotypes: located on the peritoneal surface, called superficial peritoneal endometriosis (SUP); giving rise to ovarian cysts, called ovarian endometrioma; invasion of surrounding organs, such as the bladder, vagina, ureter, or rectum, called deep infiltrating endometriosis. The American Society for Reproductive Medicine classification is based on planting location, size, and degree of surrounding adhesion, and divided into 4 stages^[2]^: stage I (minor) 1 to 5 points, stage II (mild) 6 to 15 points, stage III (moderate) 16 to 40 points, and stage IV (severe) >40 points.

It has been shown that with increasing severity of endometriosis, the live birth rate (LBR) gradually decreases after ART. Endometriosis can affect oocyte quality and embryo development potential and implantation, but the mechanism is unknown.^[3]^ A multifactor analysis showed that SUP and previous history of endometriosis surgery were both risk factors for the incidence of endometriosis-related infertility.^[4]^ For patients with stages I to II endometriosis, laparoscopic excision or ablation of endometriosis lesions can effectively improve the clinical outcome of intrauterine insemination.^[5,6]^ Nevertheless, some patients with stages I to II SUP still need in vitro fertilization (IVF) treatment. Several studies have reported that the pregnancy and implantation rates in patients with endometriosis-related infertility after IVF treatment were significantly lower, compared with patients with tubal factors or unexplained infertility.^[7,8]^

Long-acting gonadotropin-releasing hormone agonists (GnRH-a) are one of the therapeutic approaches used to treat endometriosis. Two nonrandomized trials have shown that prolonging GnRH-a down-regulation before IVF can improve pregnancy and implantation rates in patients with endometriosis undergoing IVF.^[9,10]^ Sallam et al^[11]^ conducted a meta-analysis of 3 prospective randomized trials, including 165 patients with endometriosis treated with GnRH-a for 3 to 6 months prior to IVF cycles. This meta-analysis showed that long-acting GnRH-a down-regulation significantly improved LBRs and clinical pregnancy rates (CPRs). The above studies all involved patients with fresh embryo transfer, and the subjects included patients with stages I to IV endometriosis.

Our study is the first to investigate the benefit of GnRH-a downregulation in patients with stages I to II SUP before frozen–thawed embryo transfer (FET) and whether multiple doses of GnRH-a are needed.

## 2. Methods

### 2.1. Subjects

This was a retrospective cohort study approved by the Ethics Committee of the Sir Run Run Shaw Hospital, School of Medicine, Zhejiang University, (Batch number Run Run Shaw Hospital 2022 Study No. 057) and included patients undergoing minimal and mild peritoneal endometriosis laparoscopic surgery (rAFS score, stages I–II) who underwent GnRH-a pretreatment followed by hormone replacement therapy (HRT) or HRT alone in the IVF unit of the Sir Run Run Shaw Hospital of Zhejiang University, Hangzhou, China, from March 2018 to May 2019. Inclusion criteria: (1) age 20 to 45 years old; (2) laparoscopic excision or ablation of the endometriosis lesions; (3) first transplant or second transplant cycle. Exclusion criteria: (1) intima thickness <7.5 mm or abnormal intima morphology; (2) history of endometrial hyperplasia and endometrial tuberculosis; (3) history of intrauterine adhesion surgery; (4) uterine deformity (double uterus, single horn uterus, mediastinal uterus, uterine leiomyoma, and uterine adenomyosis, etc); (5) hydrosalpinx.

**Figure 1.**
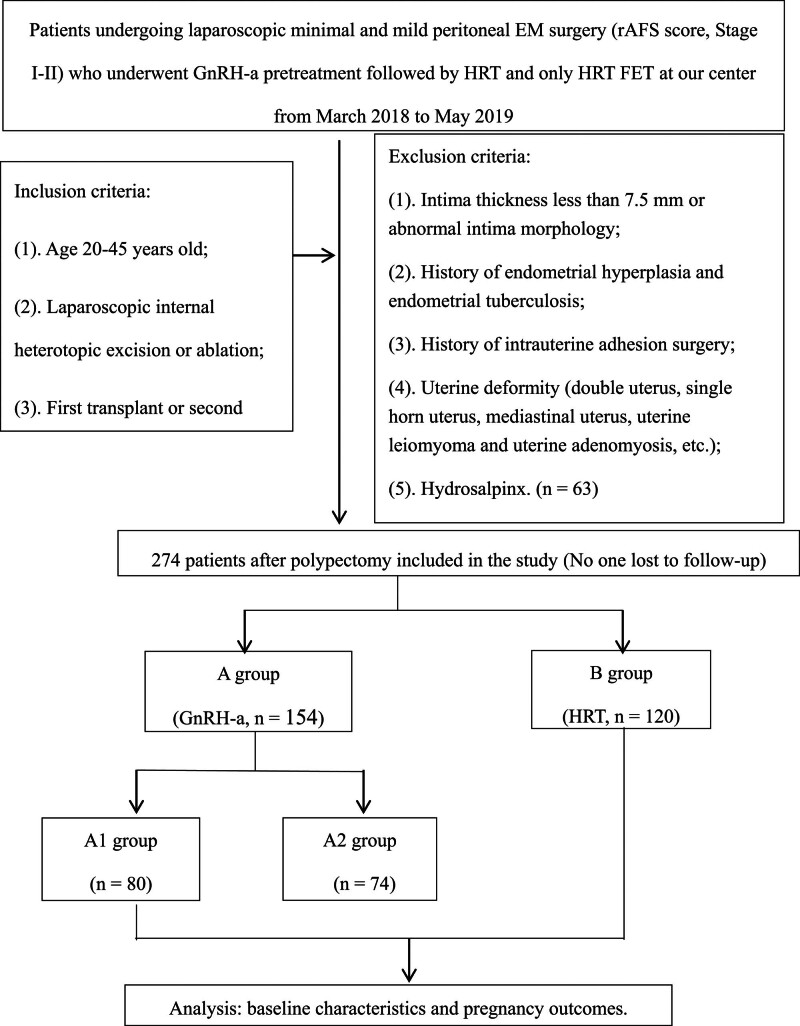
Data collection and analysis. GnRH-a: gonadotropin-releasing hormone agonist; HRT: hormone replacement therapy; FET: frozen–thawed embryo transplantation.

The enrolled patients were divided into GnRH-a–HRT (group A, n = 154) and HRT (group B, n = 120) groups. The former group was divided into single- (group A1, n = 80) and double-dose (group A2, n = 74) subgroups (Fig. 1).

### 2.2. GnRH-a and HRT protocols

The HRT protocol used an oral steroid preparation (Progynova, estradiol valerate tablets, Bayer China Co., Ltd., Guangzhou, P. R. China; or Femoston, estradiol plus dydrogesterone tablets, Abbott Healthcare Products B.V., Netherlands) at 4 to 8 mg every day on day 3 to 5 of the menstrual cycle. The GnRH-a plus HRT protocol used 3.75 mg leuprorelin (Leuprorelin Acetate Microspheres for Injection; Livzon Pharmaceutical Group, Shanghai, P. R. China) injected subcutaneously on day 2 of the menstrual cycle. A second dose of 3.75 mg leuprorelin was injected 28 days later and the HRT was started 28 days after the last injection. A transvaginal ultrasound was performed after 11 to 19 days of oral estrogen treatment. The intima needed to reach at least 7.5 mm double layer thickness without abnormal morphology, then FET was scheduled.

### 2.3. Luteal phase support and FET

Luteal phase support consisted of intramuscular progesterone in oil (Xianju Pharmaceutical Factory, Taizhou, P. R. China) at 60 mg/day for at least 2 weeks, along with oral administrations of progesterone and estradiol as above. Cleavage-stage frozen–thawed embryos were transferred at day 3 after luteal support commenced, while blastocysts were transferred at day 5. The embryos underwent vitrification, freezing, and thawing. Cleavage-stage embryos with 6 to 8 cells on day 3 and <20% fragmentation were considered “good quality”^[12]^ and blastocysts were evaluated according to a published scoring system.^[13]^

### 2.4. Follow-up after FET

Patients provided blood samples 12 days after cleavage-stage FET and 10 days after blastocyst FET. A human chorionic gonadotropin level of ≥50 IU/L was considered to indicate a biochemical pregnancy. A routine ultrasound examination was arranged 5 weeks after FET to verify clinical or ectopic pregnancy. Live birth was defined as 28 weeks of gestation or greater. Preterm birth was defined as prior to 37 weeks of gestation. Abortion included early abortion (<12 weeks of gestation) and middle and late abortion (≥12 weeks of gestation and < 26 weeks of gestation, respectively).

### 2.5. Statistical analysis

IBM SPSS Statistics software (v. 25.0; IBM Corp., Armonk, NY) was used for all statistical analysis. The measurement data were expressed as $\bar{x}\pm s$. Statistical data were expressed as percentage (%). nonparametric, Pearson Chi-square, and likelihood ratio tests were used for comparisons between groups.

## 3. Results

There were 181 patients in the GnRH-a group and 156 in the HRT group who met the inclusion criteria, and 27 patients in the GnRH-a group and 36 patients in the HRT group who met the exclusion criteria at our center from March 2018 to May 2019. In this study, 154 cases of GnRH-a (group A) and 120 cases of HRT (group B) were analyzed. Group A subgroups included 80 cases receiving 1 dose (group A1) and 74 cases receiving 2 doses (group A2) (Fig. 1).

### 3.1. Comparison of general characteristics

Blastocyst transfer in group A (10.4%) was significantly lower than that in group B (19.2%, *P* = .039). Similarly, group A2 had a blastocyst transfer rate of 8.1%, also significantly lower than group B (19.2%, *P* = .026), shown in Table 1. The number of blastocyst transfers was 1.63 ± 0.500 in group A and 1.43 ± 0.507 in group B, showing no significant difference between the groups.

**Table 1:** Characteristics of FET cycles between A versus B, A1 versus A2, and A2 versus B groups.

Group	n	Age (years)	Infertility duration (years)	Infertility		First or second cycle		Number of embryos transferred			Number of good-quality embryos	Embryos at transfer		CA125(U/mL)	Other diagnostic category		
				Primary [n (%)]	Secondary [n (%)]	First [n (%)]	Second [n (%)]	1 [n (%)]	2 [n (%)]	3 [n (%)]		Day 3 [n (%)]	Day 5 [n (%)]		None [n (%)]	Tubal [n (%)]	Male [n (%)]
A	154	32.73 ± 4.33	3.76 ± 2.61	88 (57.1)	66 (42.9)	97 (63.0)	57 (37.0)	22 (14.3)	130 (84.4)	2 (1.3)	1.22 ± 0.77	138 (89.6)	16 (10.4)	18.05 ± 8.42	97 (63.0)	55 (35.7)	2 (1.3)
B	120	32.57 ± 4.23	4.56 ± 3.12	58 (48.3)	62 (51.7)	82 (68.3)	38 (31.7)	26 (21.7)	93 (77.5)	1 (0.8)	1.08 ± 0.83	97 (80.8)	23 (19.2)	16.85 ± 7.13	60 (50.0)	51 (42.5)	9 (7.5)
A1	80	32.09 ± 3.87	3.85 ± 2.51	43 (53.8)	37 (46.2)	50 (62.5)	30 (37.5)	10 (12.5)	70 (87.5)	0	1.26 ± 0.76	70 (87.5)	10 (12.5)	16.80 ± 7.20	49 (61.3)	30 (37.5)	1 (1.2)
A2	74	33.43 ± 4.70	3.66 ± 2.74	45 (60.8)	29 (39.2)	47 (63.5)	27 (36.5)	12 (16.2)	60 (81.1)	2 (2.7)	1.18 ± 0.78	68 (91.9)	6 (8.1)	19.43 ± 9.44	48 (64.9)	25 (33.8)	1 (1.3)
Z/χ*		−0.422	−1.661	2.103		−0.921		2.611			−1.415	4.256		−0.879	9.542		
*P* *		.673	.097	.147		.357		.271			.157	.039§		.379	.008∥		
Z/χ†		−1.468	−0.905	0.783		0.017		3.491			−0.685	0.796		−1.600	0.644		
*P* †		.142	.366	.376		.896		.175			.494	.372		.110	.725		
Z/χ‡		−1.235	−1.796	0.362		0.477		1.783			−0.793	4.403		−1.614	2.673		
*P* ‡		.217	.072	.039§		.296		.410			.428	.026§		.107	.263		

There were 7 deletion of CA125 in GnRH-a group (A group) and 4 deletion of CA125 in HRT group (B group).

GnRH-a: gonadotropin-releasing hormone agonist; HRT: hormone replacement therapy; FET: frozen–thawed embryo transfer.

* A versus B.

† A1 versus A2.

‡ A2 versus B.

§ Pearson Chi-square test.

∥ Likelihood ratio test.

The combination of tubal or male factors in group A was significantly lower than that in group B (37% vs 50%, *P* = .008). The primary infertility rate in group A2 was 60.8%, which was significantly higher than that in Group B (48.3%, *P* = *.039*). There were no statistically significant differences in other basic characteristics between groups A and B, or between A2 and B. There were no significant differences in all basic characteristics between subgroups A1 and A2, shown in Table 1.

### 3.2. Comparison of pregnancy outcomes

The biochemical pregnancy, implantation rate, CPR, and LBR in group A2 were 70.3%, 46.4%, 64.9%, and 55.4%, respectively, and were significantly higher than in group B (50.8%, 35.3%, 49.2%, and 40.0%; respectively, *χ^2^* = 7.111, *P* = .006, *χ^2^* = 3.814, *P* = .033, *χ^2^* = 4.560, *P* = .023, and *χ^2^* = 4.375, *P* = .026, respectively). There were no significant differences in early abortion, middle and late abortion, premature birth, and ectopic pregnancy rates between groups A2 and B. There were no significant differences in biochemical pregnancy, clinical pregnancy, implantation, early abortion, middle and late abortion, live birth, premature birth, and ectopic pregnancy rates between groups A and B, and between subgroups A1 and A2 (Table 2).

**Table 2:** Reproductive outcomes of FET cycles between A versus B, A1 versus A2, and A2 versus B groups.

Group	n	Biochemical pregnancy rate [n (%)]	Clinical pregnancy rate [n (%)]	Implantation rate [n (%)]	Early abortion rate [n (%)]	Late abortion rate [n (%)]	Live birth rate [n (%)]	Preterm birth rate [n (%)]	Ectopic pregnancy rate [n (%)]
A	154	96 (62.3)	90 (58.4)	121 (42.0)	10 (11.1)	2 (2.2)	77 (50.0)	9 (5.8)	1 (0.6)
B	120	61 (50.8)	59 (49.2)	76 (35.3)	9 (15.3)	0	48 (40.0)	10 (8.3)	2 (1.7)
A1	80	44 (55.0)	42 (52.5)	57 (38.0)	5 (11.9)	1 (2.4)	36 (45.0)	3 (3.8)	0
A2	74	52 (70.3)	48 (64.9)	64 (46.4)	5 (10.4)	1 (2.1)	41 (55.4)	6 (8.1)	1 (1.4)
*Z/χ* ^2^ *		3.648	2.339	2.295	0.550	2.034	2.719	0.648	0.643
*P* *		.056	.126	.130	.458	.154	.099	.421	.423
Z/χ^2^†		3.818	2.420	2.070	0.050	0.009	1.665	1.345	1.473
*P* †		.051	.120	.150	.823	.924	.197	.246	.225
Z/χ^2^‡		7.111	4.560	3.814	0.005	1.936	4.375	0.003	0.030
*P* ‡		.006§	.023§	.033§	.593	.381	.026§	.591	.675

FET: frozen–thawed embryo transfer.

* A versus B.

† A1 versus A2.

‡ A2 versus B.

§ Pearson Chi-square test.

### 3.3. Logistic regression analysis

To further evaluate the influencing factors, logistic regression analysis was performed using the CPR and LBR as dependent variables, and age, GnRH-a doses, infertility duration, infertility (primary or secondary), first or second cycle, number of embryos transferred, number of good-quality embryos, embryos at transfer (day 3, day 5), CA125 level, and other diagnostic categories as independent variables.

In all patients after minimal and mild peritoneal endometriosis laparoscopic surgery (rAFS score, stages I–II), this analysis selected infertility (primary or secondary) and number of good-quality embryos affecting the LBR. FET protocols (1 dose of GnRH-a, 2 doses of GnRH-a) was not a factor in both CPR and LBR compared with HRT alone protocol (Table 3).

**Table 3:** Logistic regression analysis of the risk factors for CPR and LBR.

	CPR		LBR	
	OR (95% Cl)	*P*	OR (95% Cl)	*P*
Age	1.044 (0.977–1.115)	0.202	1.037 (0.969–1.109)	0.298
GnRH-a 1 dose	1.045 (0.565–1.933)	0.889	0.956 (0.509–1.795)	0.888
GnRH-a 2 doses	0.567 (0.292–1.101)	0.094	0.623 (0.321–1.209)	0.162
Infertility duration	1.044 (0.947–1.150)	0.390	1.073 (0.969–1.187)	0.178
Infertility (primary or secondary)	0.680 (0.389–1.190)	0.177	0.448 (0.253–0.796)	0.006*
First or second cycle	1.209 (0.676–2.162)	0.522	1.106 (0.610–2.003)	0.740
Number of embryos transferred	1.013 (0.487–2.106)	0.973	1.270 (0.598–2.696)	0.534
Number of good-quality embryos	0.620 (0.330–1.166)	0.138	0.521 (0.272–0.997)	0.049*
Embryos at transfer (D3, D5)	2.172 (0.847–5.570)	0.106	1.177 (0.459–5.570)	0.106
CA125	0.992 (0.959–1.027)	0.665	0.991 (0.957–3.019)	0.734
Combined with tubal factor	0.984 (0.258–3.750)	0.981	1.125 (0.291–4.345)	0.496
Combined with male factor	0.799 (0.214–2.989)	0.739	0.799 (0.214–2.989)	0.865

There were 3 deletion of CA125 in GnRH-a 1 dose group (A1 group), 4 deletion of CA125 in GnRH-a 2 doses group (A2 group), and 4 deletion of CA125 in HRT group (B group).

GnRH-a: gonadotropin-releasing hormone agonist; HRT: hormone replacement therapy; CPR: clinical pregnancy rate; LBR: live birth rate.

## 4. Discussion

Our study is the first to investigate the effect of pretreatment with GnRH-a in patients after minimal and mild peritoneal endometriosis laparoscopic surgery (rAFS score, stages I–II). The current results suggest that there is no significant improvement in pregnancy outcomes.

Several mechanisms are proposed for endometriosis-related infertility, including inflammatory mediators (cytokines, chemokines, and prostaglandins), peritoneal inflammation and dysfunction, pelvic anatomical malformations, and endocrine disorders.^[14,15]^ Oocytes in patients with endometriosis have low developmental potential and abnormal cytoskeletal and molecular characteristics.^[16]^ The pregnancy rate of patients receiving endometriosis patients’ donor eggs was significantly reduced.^[17,18]^ The effect of endometriosis on the endometrium is still unclear. Basic studies have shown that endometrium receptivity is reduced in patients with endometriosis, and may be related to abnormal inflammation-mediated estradiol production and progesterone resistance.^[19,20]^ Altered expression of several genes and proteins in the normal endometrium of patients with endometriosis may be related to failure of implantation.^[21]^ Other studies have shown that endometrial receptivity seems not to be affected in patients with endometriosis-related infertility.^[17,22]^ Therefore, the effect of endometriosis on endometrial receptivity is still inconclusive.

For the mechanism of GnRH-a actions, Taketani et al^[23]^ showed that the concentrations of inflammatory mediators (interleukin-1 and tumor necrosis factor) in peritoneal fluid of patients with endometriosis treated with GnRH-a were significantly lower than those in untreated patients. GnRH-a has also been reported to have immunosuppressive effects, reducing the activity of natural killer cells in patients with endometriosis.^[24]^ Ruan et al^[25]^ reported that GnRH-a treatment in patients with endometriosis could restore endometrial integrin expression (a marker of endometrial receptivity). In patients exhibiting endometriosis, the expression of endothelial nitric oxide synthase (eNOS) in endometrial glandular and luminal epithelium was higher than that in fertile women throughout the menstrual cycle, and eNOS was closely related to embryo implantation and pregnancy rates. It has been reported that ectopic endometrial eNOS levels were higher in infertile patients with stages III to IV endometriosis than in the control group (patients with cervical carcinoma in situ).^[26]^ After 3 months of GnRH-a treatment, the endometrial eNOS levels decreased in the endometriosis group.

Several clinical studies showed that the use of long-acting GnRH-a before IVF significantly improved CPR and LBR, but these studies included stages I to IV endometriosis, mainly stages III and IV.^[9–11]^ For patients undergoing IVF, a classic meta-analysis showed that the CPR and implantation rate were significantly lower for patients with endometriosis than for those with tubal factor infertility, and the higher the stage of endometriosis, the worse the pregnancy outcome.^[27]^ Further meta-analysis of patients using IVF showed that there was no difference in LBR among cases with stages I to II endometriosis compared with patients without endometriosis, while there was a lower LBR among patients with stages III/IV endometriosis.^[28,29]^ A prospective randomized trial suggested that 3 months of GnRH-a down-regulation prior to stimulation produced a significantly higher sustained pregnancy rate compared with conventional long or minimal stimulation protocols (80% vs 54%, *P* < .05). However, there were only 51 patients in this trial and no endometriosis staging.^[30]^ Another retrospective analysis showed that pregnancy rates were significantly lower in patients with mild endometriosis after IVF treatment (all type I, long protocol) compared with patients with tubular and unexplained infertility.^[31]^ All reported patients above had fresh embryo transfers.

There has been no relevant reports on whether GnRH-a downregulation is required before FET in patients with stages I to II SUP. Our study showed that there was no significant difference in pregnancy outcomes with FET between the GnRH-a down-regulation group and the HRT only group after stage I to II SUP. Although the proportion of blastocysts in the GnRH-a group was significantly lower than that in HRT group, the number of blastocyst transfers was no significant difference between the groups. And there were no differences in age, number of embryos transferred and embryo optimization rates between the groups. We found that CPR and LBR in the 2 doses of GnRH-a group were significantly higher than in the HRT only group in univariate analysis. However, infertility type (primary or secondary) and embryos at transfer (day 3, day 5) were significant differences between the groups. To control for the influence of confounding factors, Logistic regression analysis was employed. It showed that infertility type (primary and secondary) and number of good-quality embryos were influencing factors of LBR. While the number of doses (1 or 2) of GnRH-a had no significant effect on pregnancy outcomes compared with the HRT only group.

The relationship between endometriosis-associated infertility and miscarriage rate is controversial. Past studies reported increased^[32]^ or similar^[33]^ abortion rates in patients with endometriosis. There was 1 report suggesting a reduced abortion rate, which was compared with patients with unexplained infertility, but the age of patients with unexplained infertility was significantly higher than that of patients with endometriosis.^[3]^ Other research indicated that there is no significant difference in the abortion rate between spontaneous pregnancy in the same cycle after GnRH-a treatment and the general population.^[34]^ Our study suggested that there was no difference between the early abortion and late abortion GnRH-a group and the HRT group in the rate. In conclusion, it appears that GnRH-a does not increase the risk of abortion.

Strengths of the current study include that all cases of endometriosis were diagnosed by laparoscopy as exhibiting stages I to II SUP, and this is the first report investigating the role of GnRH-a descending before FET. However, there are several limitations in our study. First, this is a retrospective cohort study, which may have recall bias, and it is based on a single center. The proportion of blastocysts in the GnRH-a group was significantly lower than that in the HRT group. This should have implications for the conclusions. So further multi-center prospective research is needed. Second, the number of cases in this study is relatively small because since May 2019, our center has widely used 2-step embryo transfer (1 cleavage-stage embryo is transferred and 1 blastocyst is transferred after 2 days) which had significantly higher pregnancy rate than conventional cleaved 2 cleavage-stage embryos transfer. We did not use the data since May 2019. In addition, only 13 and 11 cases in groups A and B, respectively, underwent laparoscopic excision of endometriosis lesions with pathological diagnosis, and the other patients underwent laparoscopic ablation of endometriosis lesions.

In conclusion, for patients with stages I to II SUP-related infertility, several studies showed that laparoscopic excision or ablation of endometriosis lesions can improve their pregnancy outcomes.^[5,6]^ However, long-acting GnRH-a down-regulation before FET did not significantly increase CPR and LBR, and did not reduce the miscarriage rate in patients with stages I to II SUP. Therefore, we propose that pretreatment with GnRH-a may not be necessary before FET in patients with stages I to II SUP.

## Author contributions

**Data curation:** Jieyu Wang, Minling Wei.

**Formal analysis:** Jieyu Wang.

**Investigation:** Jieyu Wang, Aike Xu.

**Methodology:** Jieyu Wang.

**Project administration:** Jieyu Wang.

**Supervision:** Songying Zhang.

**Writing – original draft:** Jieyu Wang.

**Writing – review & editing:** Songying Zhang.
